# Editorial: Computerized Magnetic Resonance Imaging-Based Neuroimaging of Neurodegenerative Diseases

**DOI:** 10.3389/fneur.2019.00237

**Published:** 2019-03-15

**Authors:** Hans-Peter Müller, Jan Kassubek

**Affiliations:** Department of Neurology, University of Ulm, Ulm, Germany

**Keywords:** neurodegenerative diseases, neuroimaging, magnetic resonance imaging, diffusion tensor imaging, intrinsic functional connectivity MRI

While the differential diagnostic clinical work-up of patients with neurodegenerative diseases contains magnetic resonance imaging (MRI) as one of the core elements, the search of neuroimaging-based biomarkers in neurodegeneration is still an ongoing process, although tremendous advances could be reached in the last two decades. The additional value of advanced MRI, including but not limited to the potential use as a technical biological marker and phenotype characterization at the individual level with absolute quantification of regional alterations, remains a subject of investigation by multiparametric MRI protocols, including volumetry/morphometry of T1-weighted 3-D MRI, diffusion-weighted imaging (DWI), and intrinsic functional connectivity MRI (ifc MRI). For neurodegeneration (which is traditionally linked with aging), neuroimaging will be one major tool to elaborate the concept of computer-based neuroanatomy and pathoneuroanatomy, also for studies on functional reserve and compensation in aging and disease so that advanced neuroimaging (which is descriptive as a technique) has recently moved away from mere qualitative characteristics of the brain's structural and functional organization to quantitative measures and predictive models or treatment monitoring tools in disease (1). As such, there are different challenges: first, methodologically in the technical domain and second, resulting from disease-specific requirements in neurodegenerative conditions, e.g., in dementia syndromes (2), movement disorders such as parkinsonian syndromes (3), and motor neuron diseases (4).

The goal of this Research Topic was to report and reflect recent applications of multiparametric MRI to patients with various neurodegenerative diseases. Starting from applications at the group level, continuous progress of a transfer to individual diagnostic classification is reviewed and discussed. The Research Topic includes six articles and provides a synopsis of the variety of approaches of computerized MRI-based neuroimaging to neurology with the special focus on neurodegenerative diseases.

As an example for dementia syndromes, Liu et al. show changes in global brain lateralization in patients with mild cognitive impairment (MCI) and Alzheimer's disease (AD) compared to controls by resting-state functional magnetic resonance imaging (rs-fMRI). The abnormal rightward dominance observed in the patients with MCI and AD may indicate that these patients use additional brain resources to compensate for the loss of cognitive function, and the observed disappearance of the leftward laterality in the patients with AD was likely associated with the damage in the left hemisphere. That way, this study is a good example for the deepened insight into neurodegeneration on the one hand and conclusions about potential compensation by addressing differences between MCI and full dementia on the other hand. Also technically, the study shows how multisite data sharing improves the power of this kind of analyses, since the imaging data used were obtained from the Alzheimer's Disease Neuroimaging Initiative (ADNI) database (http://adni.loni.usc.edu/).

In the field of neurodegenerative multisystem disorders, Lahr et al. studied working memory-related effective connectivity in early Huntington's disease (HD) patients and pre-symptomatic HD mutation carriers by task-based functional magnetic resonance imaging (fMRI) and dynamic causal modeling. They were able to characterize effective connectivity in a WM network of HD mutation carriers by evaluating HD-related changes in the neural network underlying working memory (WM). Given that presymptomatic mutation carriers were investigated in this study, this is a blueprint of how neuroimaging in subjects with mutations for a neurodegenerative disease but without clinical symptoms can improve the understanding of disease progression and its effect on network connections in the central nerve system, with the aim to identify biomarkers and targets for potential disease-modifying interventions as early as possible.

Further studies have reviewed multifaceted aspects of computerized MRI applications to Parkinson's Disease (PD), with a focus on correlates of non-motor clinical symptoms. The structural and functional brain patterns associated with non-motor syndromes in PD were reviewed (Prell), as well as the structural and functional brain mapping correlates of impaired eye movement control in PD patients (Gorges et al.). In addition, an overview was given on the disruption of the inferior longitudinal fasciculus microstructure as assessed by diffusion tensor imaging (DTI) and its clinically significant correlation with PD symptoms, most consistently with tremor, depression/negative emotion recognition, color discrimination deficit, and cognitive decline in several domains (Haghshomar et al.). These advanced MRI studies address the deepened understanding of the underlying pathophysiological abnormalities in PD by the correlations of the clinical motor and non-motor syndrome with structural and functional brain network alterations (DTI and rs-fMRI).

As an aging-related (albeit not classically neurodegenerative) disease, Yamada et al. report on the fluid distribution pattern based on T2-weighted 3-D sequences in adult-onset congenital, idiopathic, and secondary normal-pressure hydrocephalus; they propose that adult-onset congenital normal-pressure hydrocephalus should be explicitly distinguished from idiopathic and secondary variants.

In conclusion, this Research Topic offers an overview in the field of advanced MRI-based analyses and their application to neurodegeneration and aging. These MRI-based techniques have the potential for future use in the work-up of individual patients by potentially enlarging the spectrum of non-invasive biological markers as neuroimaging-based candidate read-outs for clinical studies and by providing target information to currently available scores for longitudinal screening, Furthermore, these techniques are also prone to be used for the stratification of patients for future clinical trials in disease-modifying strategies. Further studies will complement this Research Topic, and we are looking forward to the translation of the neuroscientific evidence generated from neuroimaging progress into the clinical context.

## Author Contributions

All authors listed have made a substantial, direct and intellectual contribution to the work, and approved it for publication.

### Conflict of Interest Statement

The authors declare that the research was conducted in the absence of any commercial or financial relationships that could be construed as a potential conflict of interest.
